# Development of simple and efficient Lab-on-a-Disc platforms for automated chemical cell lysis

**DOI:** 10.1038/s41598-020-67995-3

**Published:** 2020-07-06

**Authors:** Arash Khorrami Jahromi, Maryam Saadatmand, Manouchehr Eghbal, Laleh Parsa Yeganeh

**Affiliations:** 1 Department of Chemical and Petroleum Engineering, Sharif University of Technology, Tehran, Iran; 2 Department of Electrical Engineering and Information Technology, Iranian Research Organization for Science and Technology, Tehran, Iran; 3 Molecular Bank Iranian Biological Resource Center (IBRC), ACECR Tehran, Tehran, Iran

**Keywords:** Diagnostic markers, Nucleic acids

## Abstract

Cell lysis is the most important first step for molecular biology and diagnostic testing. Recently, microfluidic systems have attracted considerable attention due to advantages associated with automation, integration and miniaturization, especially in resource-limited settings. In this work, novel centrifugal microfluidic platforms with new configurations for chemical cell lysis are presented. The developed systems employ passive form of pneumatic and inertial forces for effective mixing of lysis reagents and cell samples as well as precise fluidic control. Characterizations of the developed Lab-on-a-Discs (LoaDs) have been conducted with dyed deionized (DI) waters and white blood cells (WBCs) to demonstrate the suitability of the proposed systems in terms of mixing, fluidic control and chemical cell lysis. By making comparison between the results of a well-established manual protocol for chemical cell lysis and the proposed chemical cell lysis discs, it has been proved that the developed systems are capable of realizing automated cell lysis with high throughput in terms of proper values of average DNA yield (ranging from 20.6 to 29.8 ng/µl) and purity (ranging from 1.873 to 1.907) as well as suitability of the released DNA for polymerase chain reaction (PCR). By considering the manual chemical lysis protocol as a reference, the efficiency of the LoaDs has been determined 95.5% and 91% for 10 min and 5 min lysis time, respectively. The developed LoaDs provide simple, efficient, and fully automated chemical cell lysis units, which can be easily integrated into operational on-disc elements to obtain sample-to answer settings systems.

## Introduction

Microfluidic technologies have paved the way for miniaturization and automation of analytical protocols, enabling development of integrated point-of-care (POC) devices for early-stage diagnosis^1^. Recently, Lab-on-a-Disc (LoaD), as a relatively new type of microfluidic systems, has gained a considerable attention due to its unique features. In these systems a configuration, consisting of chambers and channels, is mounted on the surface of a disc, and the liquid is transferred through it by means of rotational forces generated by the centrifugation. The rotational forces can be harnessed for pumping and liquid handling operations. In addition, system-innate centrifugal force field decreases the number of required physical connections or pumps used in a typical microfluidic platform. Thus, the unique features result in greater complexity, sensitivity, enhanced robustness and usability of LoaD platforms compared to typical paper-based or lateral flow platforms. Collectively, LoaD platforms can be potential candidates in terms of point-of-care or point-of-use applications^2,3^.

Cell lysis is a process, in which cell membranes are disrupted with the aim of obtaining intercellular components such as DNA, RNA, proteins, and other bio-substances for further analysis. Therefore, cell lysis is considered as one of the primary steps of sample preparation in biomedical and biological applications^4^. While numerous LoaDs for various applications have been proposed, the integration of simple and efficient on-disc cell lysis has remained a challenge in the design and fabrication of sample preparation unit of LoaD platforms^5,6^.

According to previous researches, mechanical, thermal, and chemical approaches have been employed to perform cell lysis in centrifugal microfluidic platforms. Commonly in mechanical cell lysis, friction and collision between cells and beads or magnets are used to break the cell membrane^7^. However, these methods may have some downsides, including additional instrumentation and probable damage of macromoleculars^8,9^. In addition, the design and fabrication of these platforms can be challenging^7,10^. Regarding the thermal methods, the microfluidic platform should be equipped with relevant accessories for heat generation, which make the system more complex. Also heating up to high temperature can cause damage to facilities or exposed biological elements^11^. Another practical method is chemical cell lysis, in which cell membranes are disrupted by adding genuine reagents to the samples. This type of cell lysis is widely being used in protocols of the most commercial DNA extraction kits. Based on the protocols of these kits, proper mixing and subsequent incubation of lysis reagents and cell sample are performed for a certain time^11^. Thus, for implementing the chemical lysis method on centrifugal microfluidic platforms, design and fabrication of units for efficient mixing as well as precise fluidic control of samples are required. All these requirements can be readily achieved by directly designing the units on a LoaD, independent of complicated and external accessories. Thus, chemical cell lysis approach, in comparison with other types of lysis methods, has the highest potential to be integrated into LoaD platforms^12–14^.

Up to now, several innovative ideas have been applied to realize on-disc chemical cell lysis. Recently, buoyancy driven bubble was employed for mixing a cell sample and lysis reagents to perform chemical lysis on a LoaD platform. Although high cell lysis efficiency has been reported for the system, and the it has a novel mechanism and configuration, complexity of design and fabrication of the system may hamper further developments and integration of the proposed lysis unit into other on-disc units for achieving a sample-to-answer system^13^. Also, other types of automated cell lysis LoaD platforms were proposed, utilizing an integration of thermal and chemical approaches. In these studies, laser modules were used to provide desire temperature in the lysis chamber and trigger valving system. Consequently, the systems rely heavily upon external accessories, which may imposes an additional cost/complexity on performing cell lysis protocol ^11,15^.

In order to carry out on-disc chemical cell lysis, effective mixing and valving systems play the most significant roles. In the proper on-disc mixer, target cells and lysis reagents should be agitated effectively, and also the reagents are taken enough time to completely disrupt the cells’ membrane and other barriers to fully obtain intercellular substances. Subsequently, suitable valving systems should precisely control fluid flow and collect the lysate in a separate chamber for further analysis^12^. In contrast to straightforward mixing process in macroscale, the typically low flow rates and small spatial dimensions in microfluidic environments make mixing process a challenging task. Particularly, by nature, centrifugation promotes the segregation of fluids and particles with different densities, resulting in more difficult mixing operation on LoaD. Numerous mixing concepts have been applied on LoaD platform recently, including applying magnetic fields to stir liquids by magnetic beads’ movement^16,17^, convective flow induction by shake mode method^16^, liquid mixing using the moment of inertia of the liquids^18^, supply of external pneumatic pressure to agitate samples^19^, implementation of a reciprocating flow between mixing chamber and a compression chamber^20^, making the liquid streams of interest flow through specifically designed microchannels such as a serpentine channel^21^, employing highly elastic properties of embedded layers of latex to mix different liquids in a single chamber^22^, and employing buoyancy driven bubble mixing similar to macroscopic bubble columns^13,23^. As mentioned before, reliable and robust valving system is necessary for handling the reagents and processed samples on LoaDs. The valves which have been developed on centrifugal microfluidics platforms can be classified into passive and active systems, according to their reliance on external accessories. Passive valves are usually preferable due to the simplicity, being cost-effective and employing only the intrinsic centrifugation to release or retain a certain amount of liquid^24^. Siphon valves and non-siphon valves are the major subcategories of passive valves. Sharp pinning edge^25^ or hydrophobic coating^26^ are usually implemented in non-siphon valves to raise the contact angle to more than 90°. The most significant issue in designing siphon valves is engineering siphon priming. Siphon priming has been realized in varied approaches so far including hydrophilic surface treatment of siphon microchannels^27^, inducing Euler forces by applying high rotational acceleration^28^, driving the sample liquid into the siphon by creating a locally vacuum^29^, and using pneumatic action through deceleration from high to low rotational frequencies^30^. Furthermore, it seems that siphon valves triggered by pneumatic and inertial forces can be more reliable in addition to being independent of pretreatment^2,18,31,32^.

The aim of this work is to design and fabricate efficient and simple centrifugal microfluidic platforms for carrying out chemical cell lysis in a robust and automated manner. Here, for development of the chemical cell lysis discs, pneumatic and inertial forces, as two pragmatic approaches have been employed. Due to easy implementation and high efficiency of pneumatic and inertial forces, they have been applied to design passive form of mixers and valves with new configurations, which allow for effective mixing of cell sample and lysing reagents, as well as precise control of the lysate. Prior to conducting chemical cell lysis, the performance of the developed mixing and valving systems was measured. Subsequently, on-disc chemical cell lysis was conducted using a sample containing white blood cells (WBCs) and commercial lysis buffers. Finally, the lysis efficiency were measured through several relevant tests including, quantitative measurement of yields and purity of the eluted DNA samples, DNA gel electrophoresis and Polymerase chain reaction (PCR).

## Materials and methods

### Disc fabrication and experimental setup

**Figure 1:**
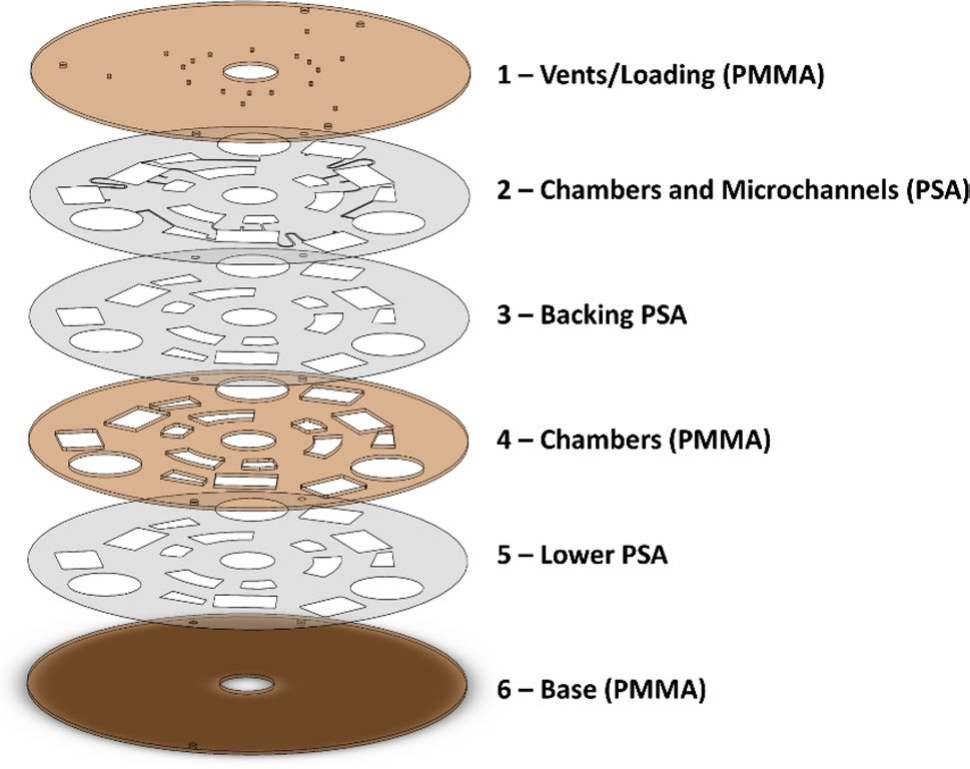
The schematic of the developed multi-layer centrifugal microfluidic platform (disc 1) which consists of PMMA layers interspersed by PSA films.

**Figure 2:**
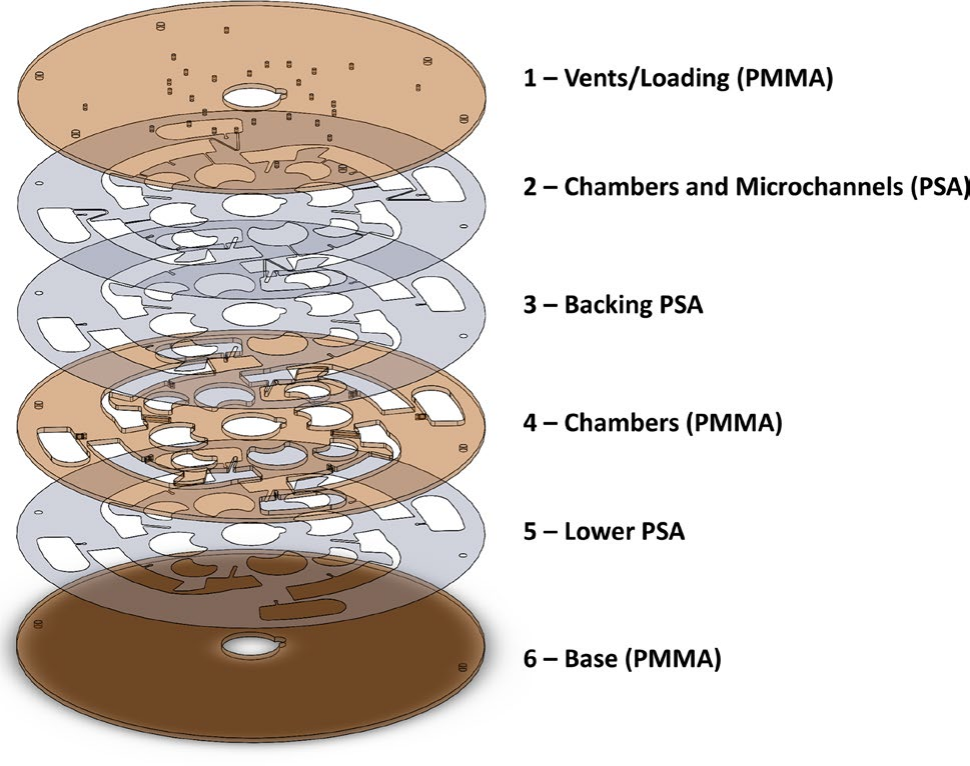
The schematic of the developed multi-layer centrifugal microfluidic platform (disc 2) which consists of PMMA layers interspersed by PSA films.

In the present work, the centrifugal microfluidic discs are comprised of three layers of Poly methyl methacrylate (PMMA, Chochen, Tainan, Taiwan) with 1.5 mm thickness, bonded together using three layers of Pressure Sensitive Adhesive (PSA, FLEXcon, USA) with 60 μm thickness. SOLIDWORKS software was used to design all pattern of elements, including microchannels, chambers, loading holes and air vents. As shown in Figs. 1 and 2, the larger elements on discs, including loading holes, air vents and chambers were created using laser cutter (Epilog Zing, USA) on PMMA layers (1, 4 and 6), while microchannels besides chambers were created using a cutter plotter (Graphtec, Japan-Graphtec CE-6000) on PSA layers (2 and 5)^33^. Furthermore, to increase hydrophilicity of the microchannels and improve optical contrast of acquired images, the microchannels can be backed by an additional PSA layer 3^2^. Figures 1 and 2 present schematic arrangements of pneumatic (disc 1) and inertial (disc 2) LoaDs, respectively. Then, the PMMA layers were washed using ethyl rubbing alcohol, and dried using an air compressor. Finally, the PMMA and PSA layers were aligned and sandwiched using a screw press to form the discs. Regarding capturing images of the spinning discs, a fully customized platform (CD Imager K1000, Key Lead Solutions Inc., San Francisco, CA, USA) was used. Discs were mounted on a computer controlled servo motor synchronized with strobe light and a stereo microscope, equipped with a short-exposure time camera. A software enables the user to determine the spinning frequencies and acceleration (or deceleration) profiles and save the captured images for image processing procedures (Fig. S1 shows an image of the experimental setup)^3^.

### Operational mechanism of the pneumatic LoaD (disc 1)

**Figure 3:**
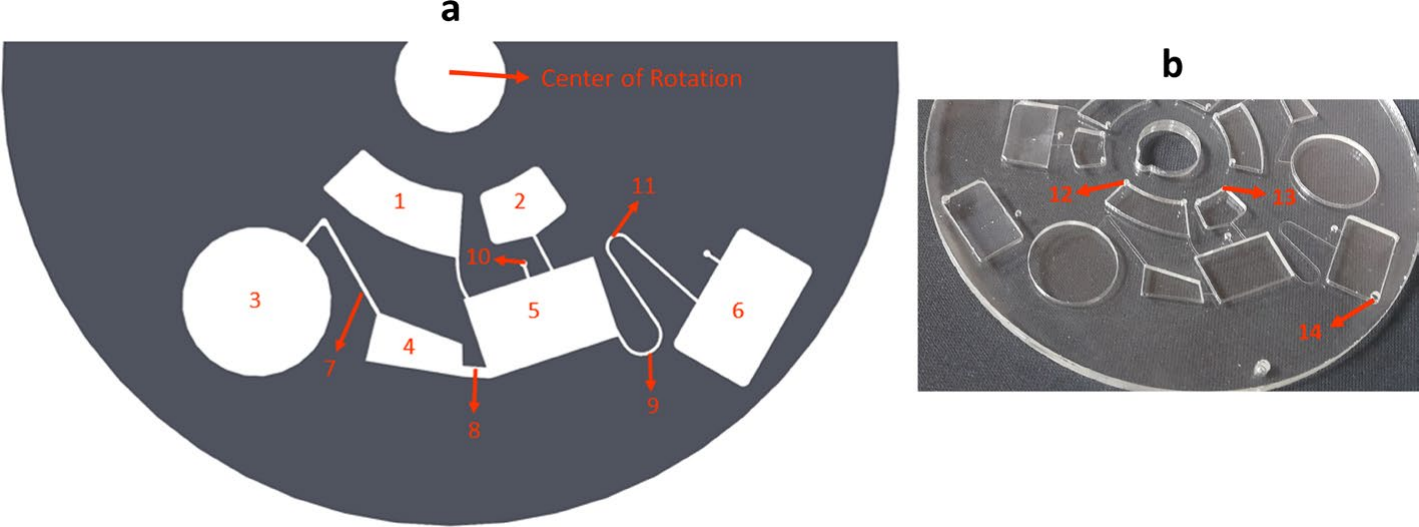
(**a**) Schematic of disc 1 operation; (1) inlet chamber (2) inlet chamber (3) pneumatic chamber (4) unvented mixing chamber (5) vented mixing chamber (6) collection chamber (7) pneumatic channel (8) mixing channel (9) pneumatic siphon valve (10) vent hole (11) crest point of the pneumatic siphon valve. (**b**) Fabricated compact disc 1; (12) Loading hole of inlet chamber 1, (13) Loading hole of inlet chamber 2, (14) lysate recovering hole of collection chamber.

The principal concept behind the design of disc 1 is to implement pneumatic energy for obtaining effective mixing and precise fluidic control of the samples, in a manner that the system is simple and independent of any external accessories. As shown in Fig. 3a, this platform includes pneumatic mixing and valving sections. First, by spinning the disc, the samples loaded into the inlet chambers are released to vented mixing chamber 5. Because of high rotational frequency, centrifugal pressure drives a proportion of the liquid into the unvented mixing chamber 4, resulting in air compression in pneumatic chamber 3. Subsequently, rapid changes in the spinning frequency leads to compression and expansion of the air in the unvented mixing chamber and pneumatic chamber. Thus, the air compression and expansion can result in mixing of the samples. Once desirable mixing has been achieved, the spin rate is abruptly reduced to a low value to collect the whole mixed sample in collection chamber 6 through the pneumatic siphon valve 9. Indeed, the abrupt deceleration can bring about sudden air expansion in the pneumatic chamber and unvented mixing chamber. As a result, the liquid is expelled from the unvented mixing chamber to the vented mixing chamber, and the liquid height in the vented mixing chamber increases above the crest point of the siphon 11, resulting in priming the siphon valve. Next, the rotational frequency is moderately pushed up to empty the content of the mixing chambers into the collection chamber. It is worth noting that the width (500 μm) and depth (60 μm) of the all channels are similar, except mixing channel 8 with 1.5 mm and 60 μm as width and depth, respectively. The larger width of mixing channel enables better transfer of samples between mixing chambers. In order to evaluate the robustness of the designed LoaD for performing different biochemical and biomedical analysis, which require a wide range of sample volume, three different types of disc 1 with capability of handling globally 180 (type A), 300 (type B), 600 (type C) µl of liquid (total volume in chambers 1 and 2) were fabricated. Figure 3b shows the fabricated compact disc type B.

### Operational mechanism of the inertial LoaD (disc 2)

**Figure 4:**
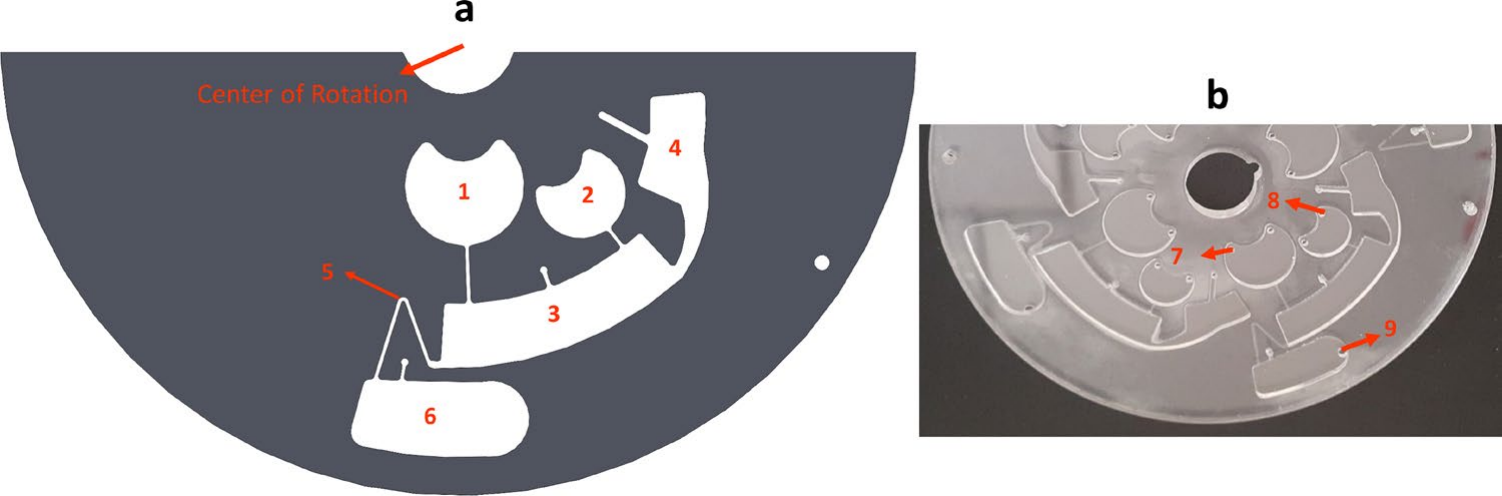
(**a**) Schematic of disc 2 operation; (1) inlet chamber (2) inlet chamber (3) mixing chamber (4) mixing chamber (5) inertial siphon valve (6) collection chamber. (**b**) Fabricated compact disc 2; (7) Loading hole of inlet chamber 1, (8) Loading hole of inlet chamber 2, (9) lysate recovering hole of collection chamber.

Similar to disc 1, the main function of disc 2 is to conduct efficient mixing and precise fluidic control, while the system is simple and independent of any external accessories. However, the general concept behind the design of this centrifugal microfluidic system is implementation of inertial force. As shown in Fig. 4a, disc 2 consists of inertial mixing and valving sections, integrated in form of a new configuration. In terms of the mechanism of the developed disc, first, by starting disc spinning, the loaded samples in the inlet chambers are released to mixing chamber 3. Then, inertial mixing is carried out through sudden deceleration of the rotating disc in counterclockwise direction. Indeed, mixing of samples between chambers 3 and 4 is performed until desirable mixing is achieved. Subsequently, the mixed sample is transported to the collection chamber 6 through several cycles, with abrupt deceleration or stop of the high-speed clockwise rotating disc. Figure 4b shows the fabricated compact disc 2.

### Mixing and valving characterization of the LoaDs

In this study, combinations of mixing and valving units have been developed in form of new configurations by employing pneumatic (disc1) and inertial (disc 2) forces. Image processing have been done to assess the mixing efficiency and valving performance. In order to highlight mixing process, yellow and blue colors were used to make two different dyed deionized (DI) water samples. The image acquisition of the liquid during the process was processed using ImageJ software for obtaining the color intensity histogram of the mixed liquid in various steps of the mixing process. In addition, the standard deviation (δ), the square root of the variance, was used for evaluating the dispersion of the data values. Standard deviation represents how much the data points differ from the mean value. Therefore, the less value of this parameter indicates the higher mixing efficiency^20^.

### Chemical cell lysis

WBCs can be potential candidates as the target cells for our lysis tests because of two reasons. First, they can be readily obtained through simple procedures from whole blood. Second, WBCs contain DNA in their nucleus, which its release can be used as a benchmark for measuring the cell lysis efficiency. To prepare the sample for the cell lysis tests, WBC pellets were isolated from 200 μl of fresh human whole blood based on typical protocols^34^. Next, the separated pellets were dissolved in 100 μl of phosphate-buffered saline (WBCs-PBS) in order to be prepared for being loaded in the inlet chamber 2. In the meantime, 200 μl and 10 μl of the lysis reagent (top blood genomic DNA extraction kit, Cat no. TGK1009, topaz gene research, Iran) and proteinase K, respectively, were loaded in inlet chamber 1. After lysate recovery from the collection chamber with a pipette, it was manually processed, according to the standard protocol of the used DNA extraction kit to obtain purified DNA. To compare the performance of the developed lysis discs with the manual protocol, as the gold-standard, the cell lysis experiments were carried out concurrently using the both proposed discs and the conventional method^15^. The manual lysis procedure includes vortexing the WBCs-PBS and the lysis reagents for 20 s at the highest setting followed by incubation of the mixture for 600 s.

### Quantitative measurement of cell lysate samples

To compare the performance of our chemical cell lysis discs with the conventional manual approach, the released DNAs in the cell lysates, obtained from both manual and automated methods, were purified, and using spectrophotometry (UV–Vis spectrophotometer device-NanoDrop, Thermo Fisher Scientific), the absorbance at 260 and 280 nm were measured to determine purity and yield (ng/µl) of the DNA. Moreover, the efficiency of the developed chemical cell lysis discs were calculated by considering the manual protocol as reference yield. The used formula has been presented below.

$$ {\text{Lysis efficiency of the disc}} = \frac{{\text{Yield of DNA obtained from the developed Load}}}{{\text{Yield of DNA obtained from manual Protocol}}} \times 100 $$

Furthermore, to check the integrity of the extracted DNA and show the quality of the lysis process in the proposed LoaD, gel electrophoresis and PCR (Bio-Rad, USA) were run for the eluted DNA in the both methods. Briefly, five µl of the eluted DNA samples, obtained from manual and automated lysis methods (disc1 and disc2), were electrophoresed on 1% (w/v) Agarose gel with 1X TAE running buffer (150 V, 50 min). Then, imaging under UV light using Gel-Doc system (Bio-Rad, USA) was carried out on DNA staining by Ethidium bromide. Moreover, the PCR amplification of human mitochondrial Cytochrome C oxidase subunit I (COI) gene was carried out by thermal cycler (Bio-Rad, USA). PCR was performed as below; reaction mixture contained 2 µl of template DNA (50 ng), 1 µl of forward primer (LCO1490: 5′-GGTCAACAAATCATAAAGATATTGG-3′) and 1 µl of reverse primer (HCO2198: 5′-TAAACTTCAGGGTGACCAAAAAATCA-3′) (10 PM), 12.5 µl of 2× master mix (Thermo fisher scientific) and 9.5 µl of deionized water. Fragment amplification was done by initial denaturation at 94 °C for 3 min followed by 5 cycles of 94 °C for 45 s, 45 °C for 90 s, and 72 °C for 60 s, 25 cycles of 94 °C for 45 s, 51 °C for 90 s, and 72 °C for 60 s, final extension at 72 °C for 5 min. Five µl of PCR products was electrophoresed on 1% (w/v) Agarose gel with 1X TAE running buffer (150 V , 50 min), PCR product staining by Ethidium bromide and imaging under UV light with Gel-Doc system (Bio-Rad-USA).

## Results and discussion

### Evaluation of mixing and valving systems

**Figure 5:**
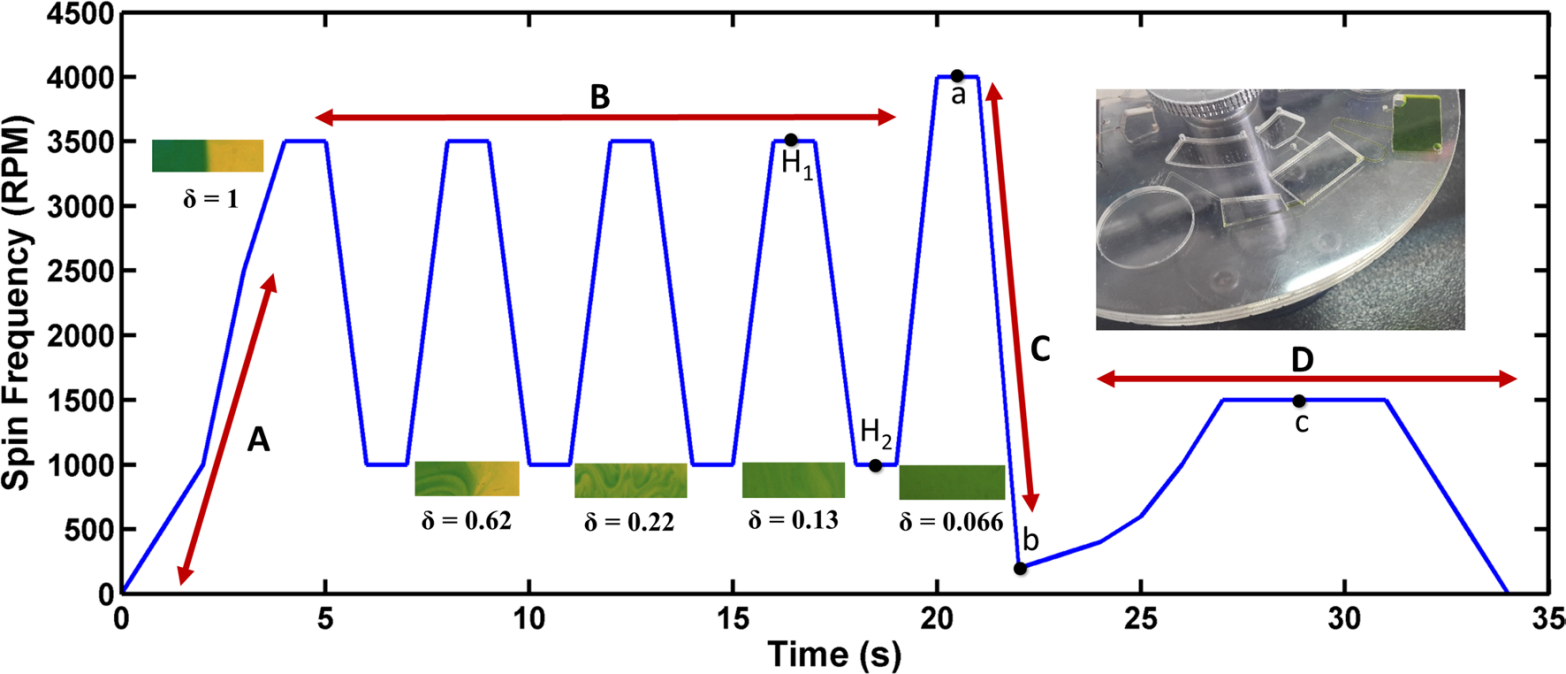
Spin protocol and mixing performance of disc 1 (type B). (A) Sample introduction, (B) reciprocating, mixing (C), siphon priming (D) sample collection (H_1_) a point at the highest rotational frequency during mixing cycles, (H_2_) a point at the lowest rotational frequency during mixing cycles, (a) a point in which the level of liquid is below the crest point, (b) a point in which siphon primes, (c) a point in which the liquid begins to transfer to collection chamber.

To measure the performance of the mixing and valving systems of the discs, the developed discs were run for dyed deionized (DI) water. However, owing to the fact that in our designs the only driving force for handling liquids are rotational forces, one of the main challenges is to obtain an optimized spin protocol for the discs. Regarding disc 1, in which pneumatic force is implemented in a passive form, four steps have been determined to realize its complete function (Fig. 5). In step A (sample introduction), the loaded initial samples (lysis reagents/ WBCs-PBS or dyed DI water) are driven to mixing chambers by centrifugal force. Then, in step B (reciprocating mixing), the reciprocating spinning profile results in the pneumatic mixing. Subsequently through exerting high deceleration in spin rate, performed in step C (siphon priming), the pneumatic siphon is primed. Finally, in step D (sample collection), the whole processed sample (lysate or mixed liquids) is emptied into the collection chamber. After obtaining an optimized spin protocol for the discs, image processing was carried out to investigate the mixing efficiency and valving performance.

**Figure 6:**
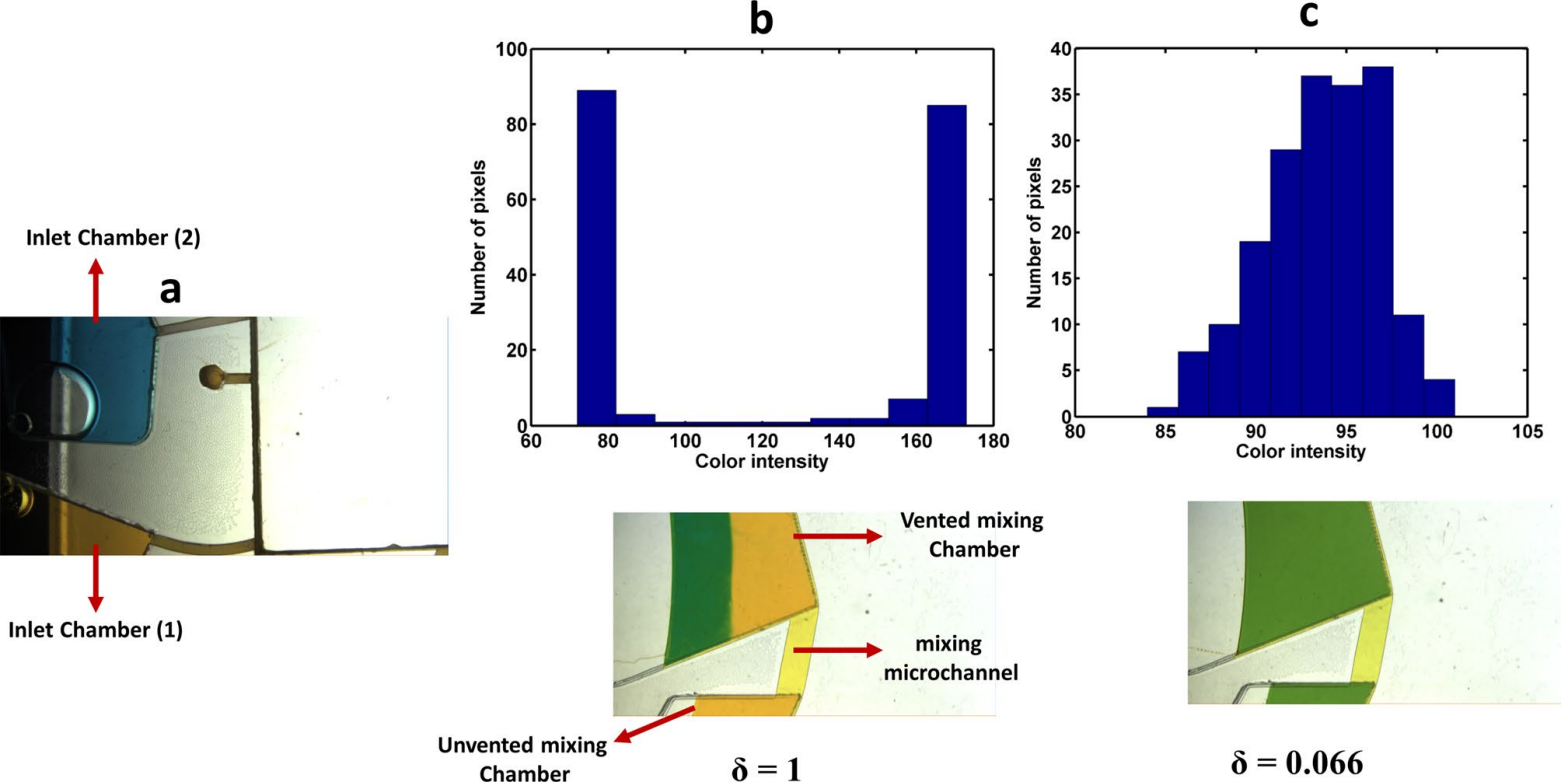
Evaluation of the mixing performance using intensity histogram for disc 1 (type B); (**a**) loaded samples in the inlet chambers, (**b**) 2D color intensity histogram obtained after samples introduction, (**c**) 2D color intensity histogram obtained after fourth cycle.

For instance, as Fig. 5 depicts, a homogenous mixture was achieved with disc 1 (type B) after four cycles. As the calculated values of the standard deviation (δ) approach to zero, a more homogenous mixture is achieved. The reduction of δ from 1 (after introduction of samples to the mixing chambers) to 0.006 (after fourth cycle) shows outstanding performance of the pneumatic mixing system. In addition, at the end of fourth cycle the color intensity histogram of the mixing process was determined (Fig. 6). The wide distribution of the color intensity of pixels in Fig. 6b, which is represented by two peaks in the histogram, as well as the high standard deviation show unmixed samples after the samples introduction step. Along the mixing progress, peak width of the pixel intensity in the histogram diminishes, and consequently the value of standard deviation decreases. Then, as can be seen in Fig. 6c, after fourth cycle a homogenous mixture was achieved. To provide more details about the function of pneumatic mixing, the capability of pneumatic force has been demonstrated in Fig. S2. Indeed, the sudden disc deceleration causes the expansion of compressed air, which leads to change of the liquid level. H_1_ and H_2_ (which are shown in Fig. 5) can represent empty volume of the unvented mixing chamber at the time of high (3,500 rpm) and low (1,000 rpm) rotational frequencies, respectively. Therefore, the several changes in the liquid level can bring about the desirable agitation.

Handling and collection of the mixed sample is a crucial part of this work. Fig. S3a shows the pneumatic siphon valve at high rotational frequency, when the level of liquid is below the crest point (state a in Fig. 5). Then, as seen in Fig. S3b, the abrupt deceleration leads to a sudden air expansion in the pneumatic chamber which consequently expels the liquid from the unvented mixing chamber and the siphon primes (state b in Fig. 5). By increasing the rotational frequency, as seen in Fig. S3c, the liquid begins to transfer to collection chamber (state c in Fig. 5), and finally the whole sample is collected in the collection chamber. These images prove that the pneumatic siphon valve is able to handle the whole sample by employing an appropriate spin protocol.

**Figure 7:**
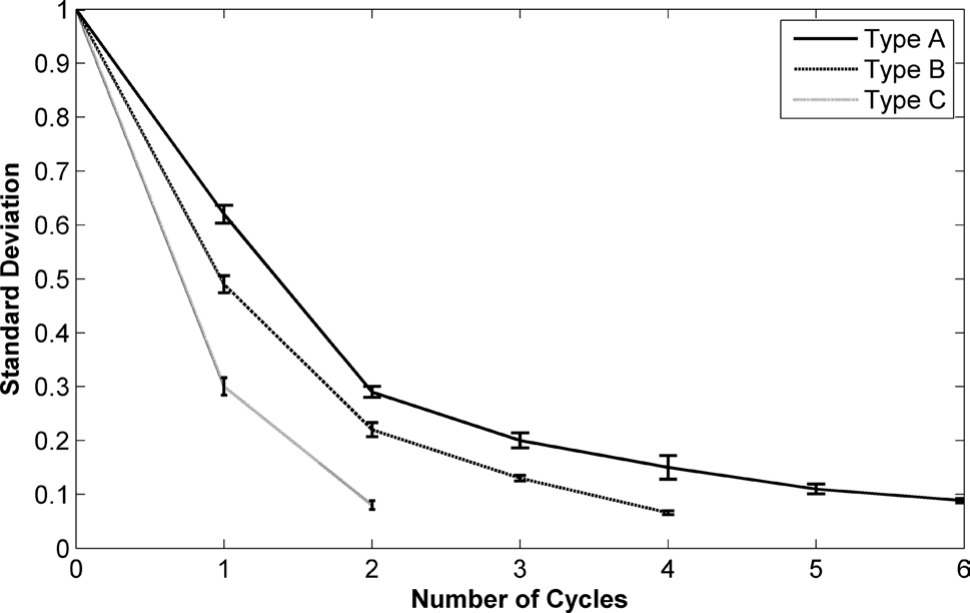
Demonstration of the standard deviation values for the mixed liquid in various cycles for the different types of disc 1 (n = 3).

To make comparison between the mixing performance of the three different types of disc 1 (types A, B and C), the standard deviation values of the mixed liquids after each rotation cycle has been plotted in Fig. 7. By increasing the volume of mixing chambers, in relation to the different types of disc 1, the results showed that, at similar spin protocol, through fewer cycles the fully mixed sample (0.1 > δ) can be obtained. Consequently, 6, 4 and 2 cycles resulted in fully mixed samples in types A, B and C respectively. However, the performance of pneumatic siphon valve is independent of the chambers’ volume, and in the all types the whole mixed sample could be transferred to the collection chamber. Therefore, our developed chemical cell lysis disc can be tailored to operate with various volumes of samples with only slight design modifications.

**Figure 8:**
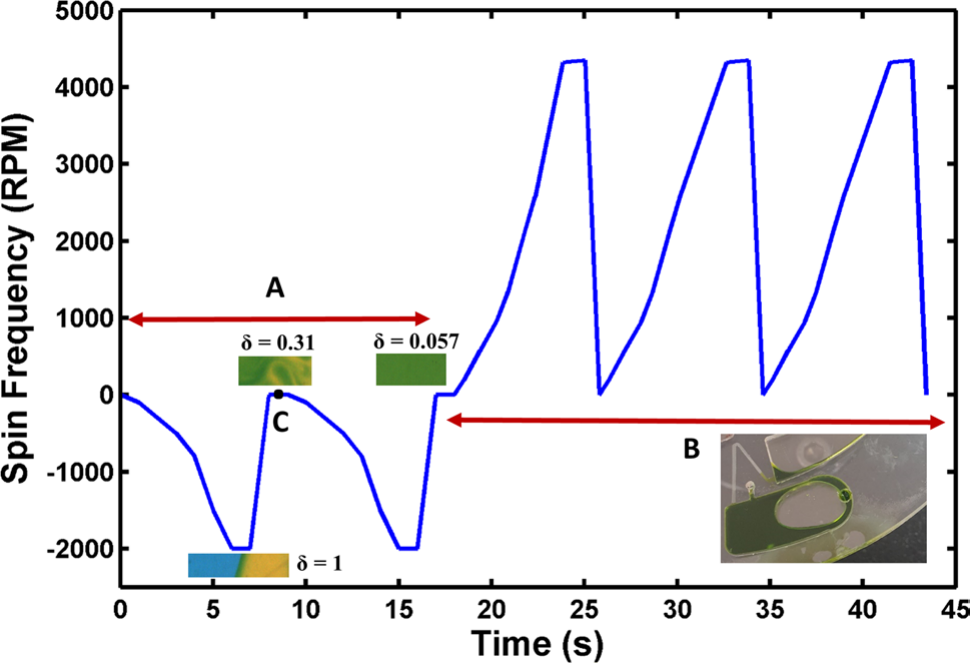
Spin protocol and mixing performance of disc 2. (**A**) counterclockwise reciprocating mixing, (**B**) collection through clockwise cycles, (**C**) a point in which the disc is stopped while it was rotating in counterclockwise direction.

In terms of disc 2, in which inertial force is implemented in a passive form, the loaded samples in the inlet chambers are released to mixing chambers by starting disc spinning as shown in Fig. 8. Then, according to the obtained spin protocol, counterclockwise reciprocating mixing (step A) is performed for two cycles; subsequently, the mixed sample is transferred to the collection chamber through three clockwise cycles (step B).

The effect of moment of inertia in mixing process is demonstrated in Fig. S3. This figure shows a state in which the disc is stopped while it was rotating in counterclockwise direction (this condition has been depicted in the Spin protocol in Fig. 8). Therefore, by applying inertial force in form of the developed configuration a considerable amount of liquid can be transferred.

**Figure 9:**
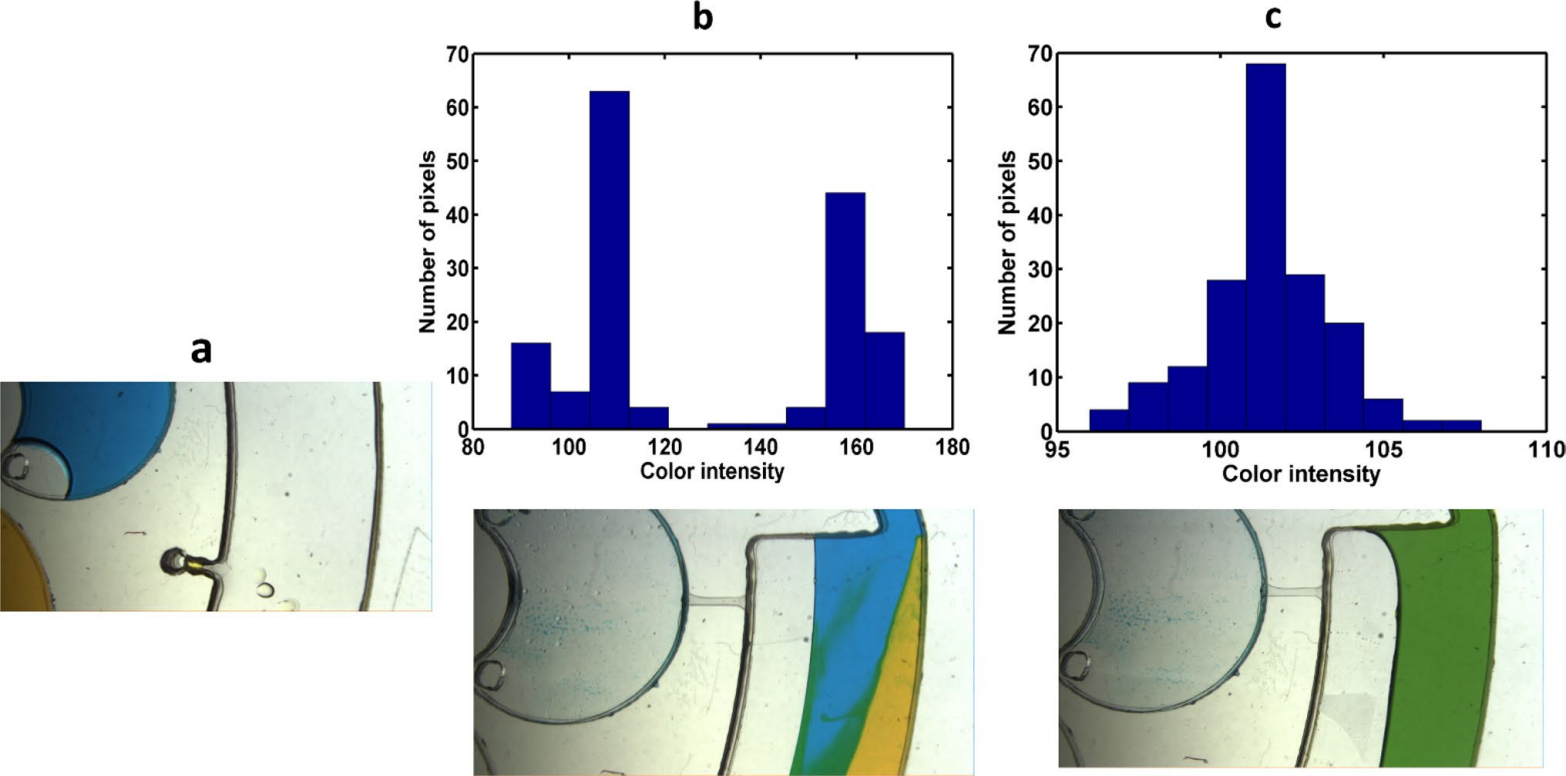
Evaluation of the mixing performance using intensity histogram for disc 2; (**a**) loaded samples in the inlet chambers, (**b**) 2D color intensity histogram obtained after samples introduction, (**c**) 2D color intensity histogram obtained after fourth cycle.

**Figure 10:**
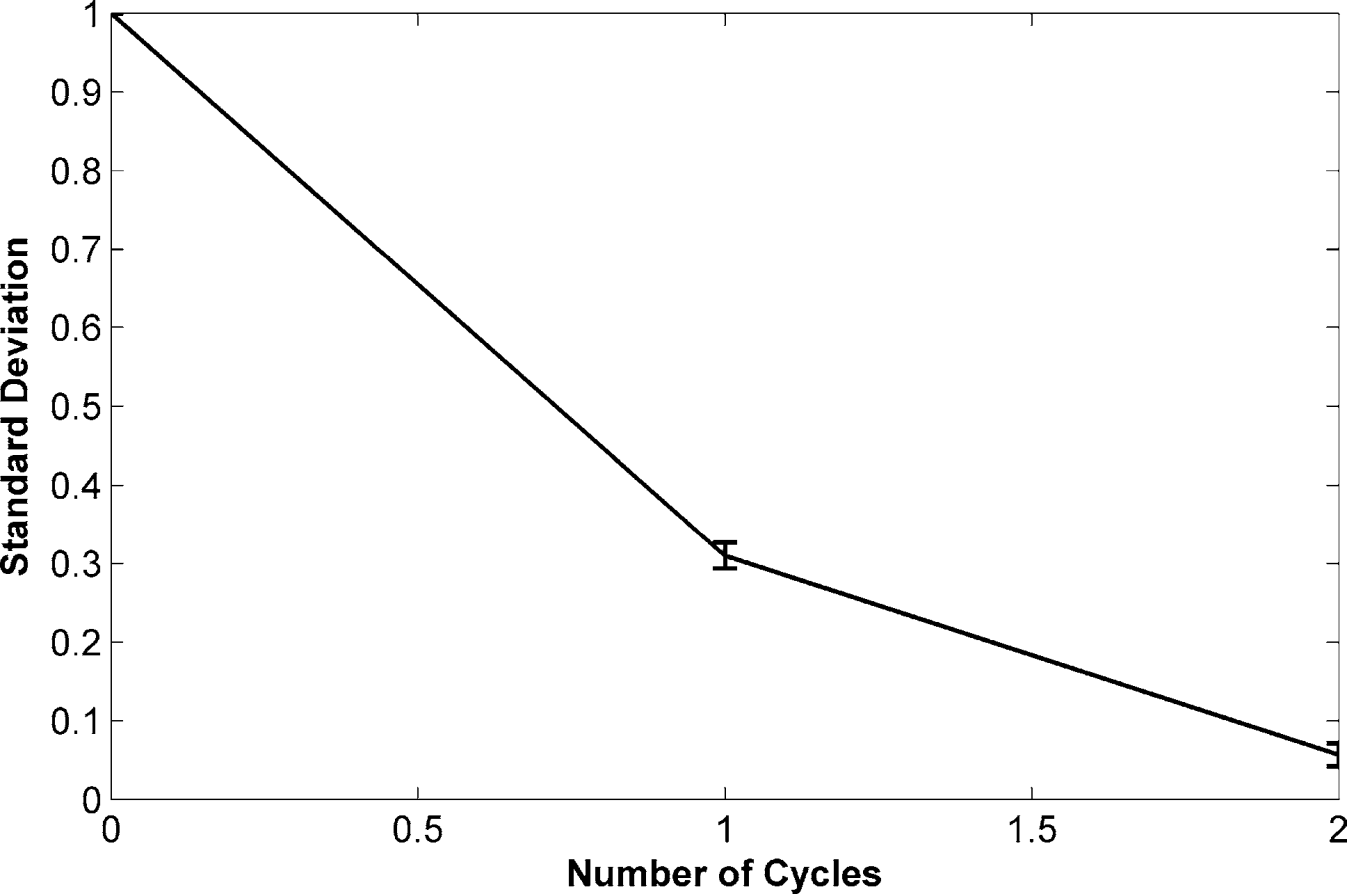
Demonstration of the standard deviation values for the mixed liquid in various cycles for disc 2 (n = 3).

As shown in Figs. 9 and 10, similar to the analysis of mixing system for disc 1, color intensity histogram and standard deviation values were determined for processed liquids in disc 2.

Figure S5 demonstrates the function of the developed inertial siphon valves, which can transfer the processed sample through three cycles, which can vary depending on the value of deceleration.

Collectively, the evaluations prove that disc 2 is superior to disc 1 in terms of performing mixing. However, due to the different mechanism of pneumatic and inertial siphon valves, the latter probably cannot transfer the whole processed sample and it is more likely that an insignificant amount of liquid remains in the mixing chamber of disc 2. In addition, generally inertial siphon valve requires higher levels of deceleration (more than threefold) for its function compared to pneumatic siphon valve. In our work the value of deceleration needed for inertial siphon valve is more than three times as high as the value required for the pneumatic siphon valve. But, it is notable that this ratio can vary depending on the physical characteristics of the valves.

### On-disc chemical cell lysis

One of the main applications of the proposed discs would be to perform chemical cell lysis in an automated way. Up to now, pneumatic and rotational forces have been employed for performing chemical cell lysis on disc^13^. However, some flaws in configuration of the disc's elements, including chambers and microchannels, brought about relatively ineffective cell lysis. The relative lower efficiency of on-disc chemical cell lysis using pneumatic and rotational forces may stem from two main origins. The former is most likely due to the low intensity of mixing in some configurations. For example, the Euler force is not strong enough to bring about a sloshing of the liquids and the internal fluid flows are not adequate to cause rigorous mixing of cell sample and lysis reagent. The latter would be sedimentation of cells, which should be lysed, in both chambers and the connecting microfluidic channel due to high rotational frequencies. Therefore, in the present work, some modifications have been made to conventional disc architectures in order to create more effective agitation and eliminate sedimentation effect. Regarding employing rigorous mixing, the image processing has demonstrated that desirable mixing can be obtained through effective implementation of inertial and pneumatic forces in passive forms. Moreover, to reduce the possible sedimentation effect, connecting channels between mixing chambers and also between collection and mixing chambers should connect the elements from the points located in the relative higher radial distance (Figs. 3 and 4). This kind of arrangements were applied to both disc 1 and disc 2. The arrangement leads to the fact that the cells can be effectively transferred between mixing chambers and totally collected in the collection chamber after entirely emptying the mixing chambers by means of the pneumatic and inertial siphon valves. In this way, the risk of sedimentation of cells to the bottom walls of the mixing chamber can be substantially eliminated. Also, if there are any intact cells in the lysate after the mixing process, they will be lysed during transferring the lysate to the collection chamber and the following short-time incubation.

**Figure 11:**
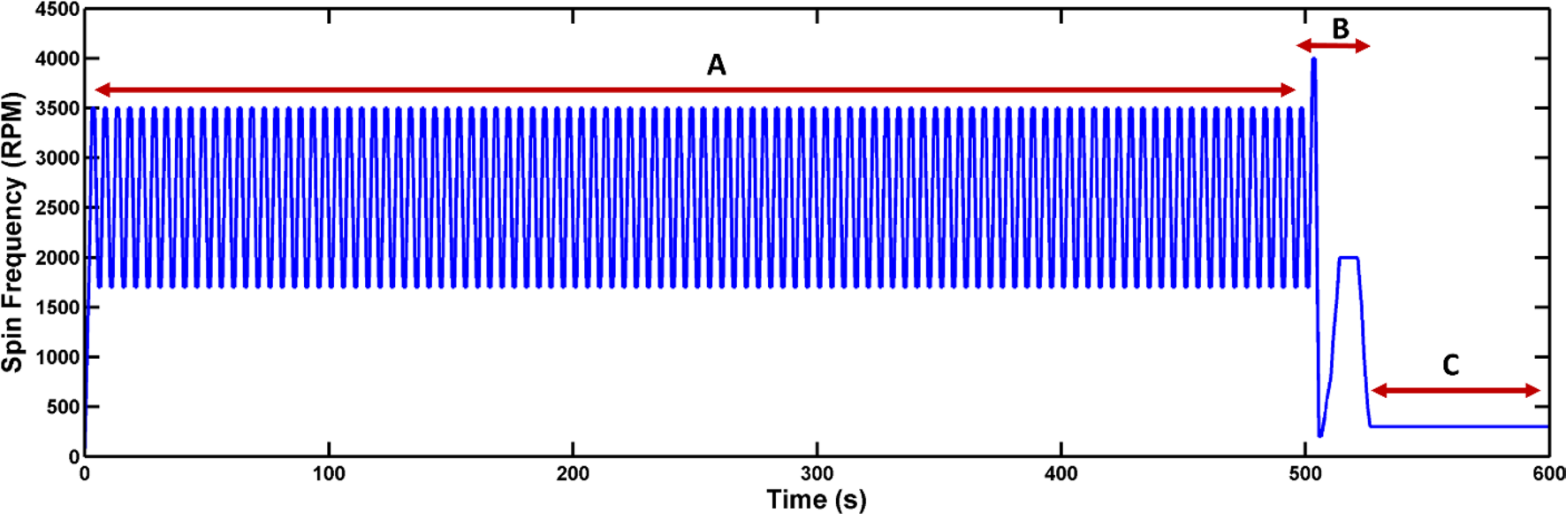
Spin protocol for cell lysis on disc 1. (**A**) Mixing of WBC sample (100 µl) and lysis buffer (190 µl)/Proteinase K (10 µl), (**B**) collection of lysate using pneumatic siphon, (**C**) incubation of the lysate.

**Figure 12:**
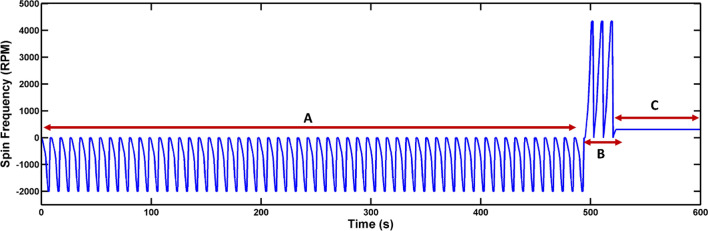
Spin protocol for cell lysis on disc 2. (**A**) Mixing of WBC sample (100 µl) and lysis buffer (190 µl)/Proteinase K (10 µl), (**B**) Collection of lysate using inertial siphon, (**C**) Incubation of the lysate.

Since mixing and collection of samples are primary procedures for performing on-disc chemical cell lysis, the trend of rotational frequency for the cell process should be similar to the obtained spin profiles for the developed discs. However, based on protocol of the used commercial kit (600 s incubation as well as 20 s vortex), more time is needed for effective interactions between the WBCs and lysis reagents. Therefore, the operation time of the discs should be extended for lysate preparation. Approximately 500 and 100 s as extended discs’ operation time were considered for the mixing process and collectively transferring and incubation of lysate in collection chamber respectively. Thus, WBCs-PBS and the lysis reagents were mixed for 500 s, followed by 100 s for transferring and incubation of lysate. If any cells remain intact in mixing process, they are lysed during incubation at low frequency (300 rpm) in the collection chamber. Figures 11 and 12 illustrate the used spin protocol for chemical cell lysis on discs 1 and 2, respectively. In these figures, in steps A, B and C, mixing of lysis buffer and WBC sample, siphon priming and incubation are performed, respectively.

According to the used DNA extraction protocol, the required volume of cell sample (100 µl of PBS-WBC) and lysis reagents (190 µl of lysis buffer and 10 µl of proteinase K) match the volume capacity of type B of disc 1 and also disc 2. Therefore, the chemical cell lysis was performed by means of these two types of discs.

#### Quantitative measurement of the cell lysate

According to the performed image processing, the developed discs have shown outstanding performance in terms of mixing of samples. However, a certain level of homogeneity in the grey scale distribution cannot capture the whole complexity of the actual mixing process, required for biochemical applications such as cell lysis. Thus, to assess the efficiency of the nuclear lysis, after recovering the lysate from the discs with a pipette, DNAs in the cell lysates were manually purified using a DNA extraction kit^35^. It is obvious that the quantities of purity and yield of the extracted DNA are indicative of the performance of the suggested chemical cell lysis discs. Indeed, proper values of the DNA purity, ranging from 1.8 to 2, and cell lysis efficiency (the ratio of the LoaD DNA yield to the manual protocol DNA yield, as the gold-standard) can demonstrate capability of the proposed systems to effectively perform chemical cell lysis^35^. In addition, to show the importance of the required time for interaction between lysis reagents and WBCs, the 10 min lysis time was halved to 5 min, and same quantitative measurements were performed for this case. To confirm reproducibility, 7 and 5 replicate experiments were conducted for 10 and 5 min lysis time, respectively (Table 1). Regarding 10 min lysis time, the average purity of DNA obtained from the lysis discs and the manual protocol (spin column) were 1.891 and 1.917 respectively, and the mean yield of the released DNA were 29.9 ng/µl and 31.3 ng/µl, respectively (Table 1a). Thus, based on the defined lysis efficiency for the developed discs, this value is 95% and 96% for disc 1 and disc 2, respectively. However, the average yield of extracted DNA achieved by manual protocol and automated lysis discs decreased to 22.9 ng/µl and 20.9, respectively for 5 min lysis time (Table 1b). Subsequently, the efficiency was determined 92%, 90% for disc 1 and disc 2 respectively. These high values of the developed discs’ efficiency demonstrate that the proposed configuration for chemical cell lysis can be pragmatic approaches, which may overcome shortcomings of previous similar works, employing inertial and pneumatic forces^11,13–15^.

**Table 1:** DNA yield, DNA purity, and lysis efficiency of the discs for (a) lysis time = 10 min, (b) lysis time = 5 min.

Sample number	Disc 1		Disc 2		Manual protocol	
	Purity	Yield (ng/µl)	Purity	Yield (ng/µl)	Purity	Yield (ng/µl)
**(a) Lysis time = 10 min**						
1	1.6780	26.4	1.8250	37.6	1.896	38.8
2	1.8950	30.5	1.6980	28.2	1.795	28.2
3	1.7540	28.5	1.9890	26.5	1.989	30.5
4	1.9920	34.8	2.0150	26.9	1.958	32.1
5	2.0180	31.2	1.7787	27.5	1.912	29.3
6	1.9340	29.7	1.7920	32.2	1.792	26.9
7	2.0800	27.3	2.0180	30.3	2.08	33.6
Average	1.907	29.8	1.874	29.9	1.917	31.3
Efficiency	95%		96%		100% (reference)	
Data standard deviation	0.1450	2.7974	0.1310	3.9570	0.1036	3.9954
**(b) lysis time = 5 min**						
1	1.809	22.8	1.8390	18.9	1.723	21.3
2	2.111	25.7	1.7010	21.1	1.921	27.2
3	1.672	16.9	2.0800	20.5	1.825	17.5
4	1.874	21.1	1.7580	19.4	1.879	22.7
5	1.933	19.1	1.9910	22.9	2.101	26.1
Average	1.880	21.1	1.8738	20.6	1.889	22.9
Efficiency	92%		90%		100% (reference)	
Data standard deviation	0.1616	3.3796	0.1587	1.5710	0.1394	3.8882

It is worth noting that owing to the considerable reduction in DNA yields, it appears that the lysis time less than 5 min may not be feasible and practical for clinical uses. Although, compared to type B of disc 1, fully mixed sample can be obtained more efficiently by means of disc 2 (through fewer cycles), it can be clearly seen that there are insignificant differences between the performances of the developed discs for automated chemical cell lysis. This observation shows that the both centrifugal microfluidic platforms (disc 1 and disc 2) are able to provide an effective interaction between WBCs and lysis reagents for cell disruption for the duration of 10 or 5 min.

**Figure 13:**
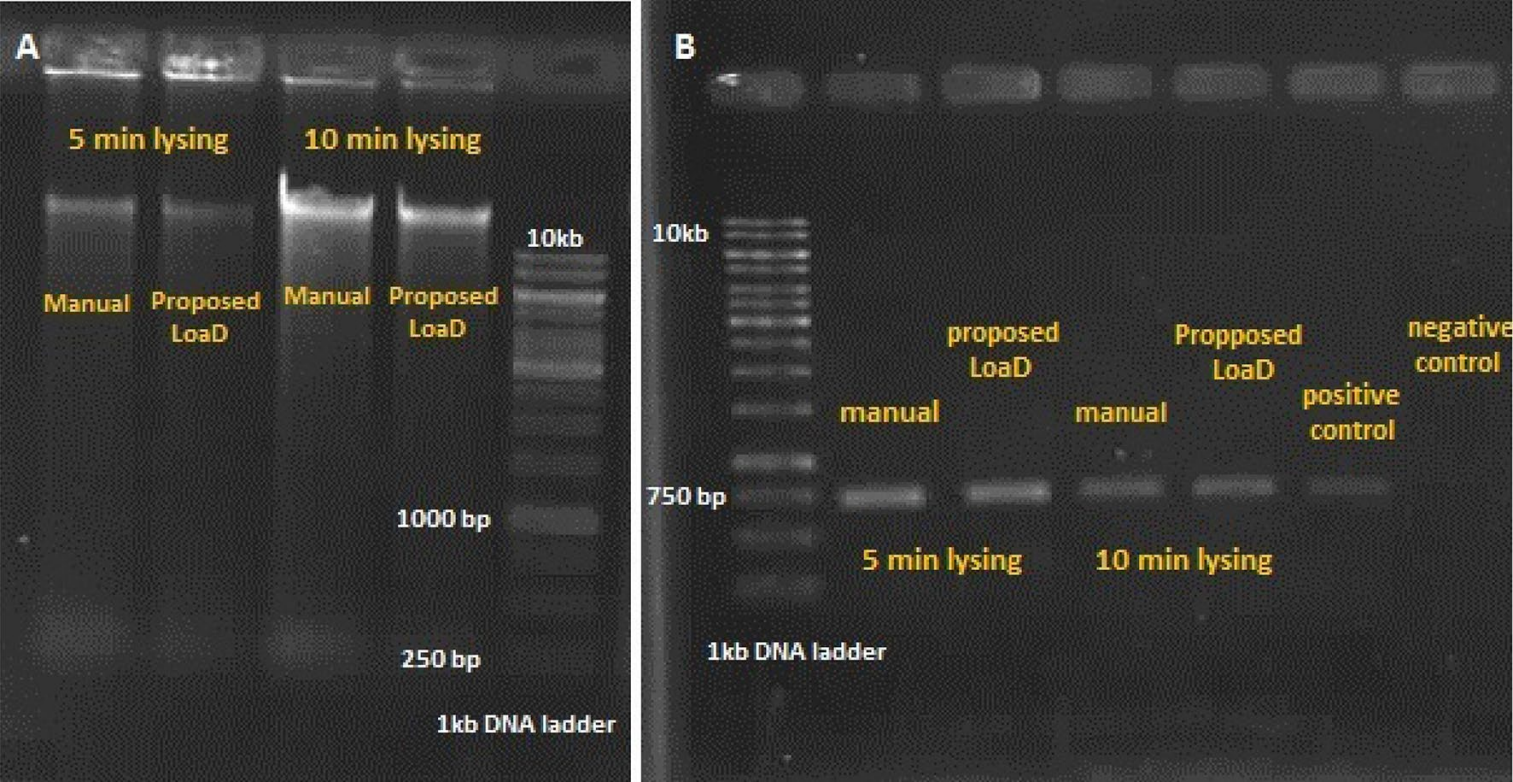
PCR and Gel electrophoresis of the purified DNA obtained from disc 1 and manual protocol. (**A**) DNA bands and (**B**) PCR products.

To evaluate the integrity of the extracted DNA in the lysate, gel electrophoresis was performed on the eluted DNA samples, obtained from manual and automated lysis methods (disc 1 and disc 2). As can be seen in Fig. 13, sharp DNA bands were found, while no band appeared at any other positions. It can be concluded that DNA prepared by the developed chemical cell lysis discs was neither damaged nor fragmented.

To assess the clinical applicability of the developed chemical cell lysis discs, a PCR amplification and visualization was conducted for the purified DNA, obtained from on-disc and manual lysis protocols (Fig. 13). Due to the fact that results of gel electrophoresis and PCR for the DNAs, obtained from disc 1 and disc 2, seemed very similar, only the results of disc 1 have been compared with the results of the manual protocol in Fig. 13. As the results demonstrate, the target PCR bands can be clearly seen. Therefore, the developed chemical cell lysis discs show promise for clinical applications.

Generally, in addition to cell lysis automation on LoaD platforms, the developed systems showed comparable results with the conventional commercially chemical cell lysis protocols. It is worth noting that the modifications do not add complexity to the design and fabrication of the discs while enhance the loss in DNA yields.

## Conclusion and future perspective

Here, in an application oriented way, two different centrifugal microfluidic platforms have been developed for performing automated chemical cell lysis. Pneumatic and inertial forces, in passive forms, have been employed for carrying out mixing cell samples and lysis buffers as well as multidirectional pumping of liquids in form of siphon valves. In this work, it has been attempted to eliminate shortcomings in configurations of previous cell lysis discs. In other words, novel arrangements of elements (chambers and microchannels) have been suggested to deal with both low level of liquid agitation and sedimentation of cells, which have been major challenges for on-disc chemical cell lysis. In addition, the proposed arrangement can be a viable alternative to current complicated LoaDs platforms used for mixing and precise fluidic control. The high efficiency of mixing and valving systems was proved by means of image processing. Indeed, the both types of the developed discs after a few mixing cycles (ranging from 2 to up to 6 cycles) can make the samples fully mixed. Also, approximately whole processed sample can be transported using developed multidirectional pumping systems, which are called inertial and siphon valves.

Moreover, quantitative measurements of the cell lysate, including measuring of yields and purity of the eluted DNA samples, DNA gel electrophoresis and PCR, demonstrate the suitability of the developed chemical cell lysis discs. Because, the results obtained from the developed LoaD are totally comparable with the manual lysis procedure. The purified DNA obtained from the both types of developed chemical cell lysis discs possessed high purity (ranging from 1.873 to 1.907). Nuclear lysis of WBCs, separated from human whole blood, in 10 min using disc 1 and disc 2 lead to average yield of 29.8 ng/µl and 29.9 ng/µl purified DNA respectively, and these values decrease to 21.1 ng/µl and 20.6 ng/µl by reducing lysis time to 5 min. Theses yield values can be sufficient for clinical uses. Additionally, by considering the manual protocol as a reference yield, the average efficiency of the discs are 95.5% and 91% for 10 min and 5 min respectively, which show great performance of the developed LoaDs. Collectively, although disc 2 shows superiority in terms mixing at initial time compared to disc 1, they have same lysis performance due to the required extended time for cell lysis.

Therefore, all of the required measures, which should be taken into account to ensure that the developed discs are robust platforms for chemical cell lysis, have been employed in this work. Furthermore, although our systems may not be considered as real point-of-care platforms, the proposed configurations have a great potential to be easily integrated into relevant on-disc operational elements for obtaining complete sample-to-answer systems. This is because of ease of design and fabrication of the developed centrifugal platforms as well as high efficiency of the automated chemical cell lysis discs. For example, due to the fact that reagents mixing and sequential fluidic control are highly required in automated DNA extraction and purification, the developed arrangement of the inertial and pneumatic mixing and also valving systems can be employed with some simple design changes. In terms of our future work, by using obtained technical knowledge about mechanism of pneumatic and inertial systems on centrifugal microfluidic platforms, we will develop a LoaD for DNA extraction and purification from whole blood.

## Supplementary information

Supplementary information
