# Supplementary material for: Development of simple and efficient Lab-on-a-Disc platforms for automated chemical cell lysis
Source: Sci Rep. 2020 Jul 6;10:11039. doi: 10.1038/s41598-020-67995-3 (PMC7338454; doi:10.1038/s41598-020-67995-3)
Supplement: Supplementary file 1 — Supplementary information [file 41598_2020_67995_MOESM1_ESM.docx]

**Development of Simple and Efficient Lab-on-a-Disc Platforms for Automated Chemical Cell Lysis (Supplementary)**

**Arash Khorrami Jahromi^1^, Maryam Saadatmand^*1^, Manouchehr Eghbal^2^**, **Laleh Parsa Yeganeh^3^**

1 Department of Chemical & Petroleum Engineering, Sharif University of Technology, Tehran, Iran.

2 Department of Electrical Engineering and Information Technology Iranian Research Organization for Science and Technology, Tehran, Iran.

3Molecular bank Iranian Biological Resource Center (IBRC), ACECR Tehran, Iran

*m.saadatmand@sharif.edu


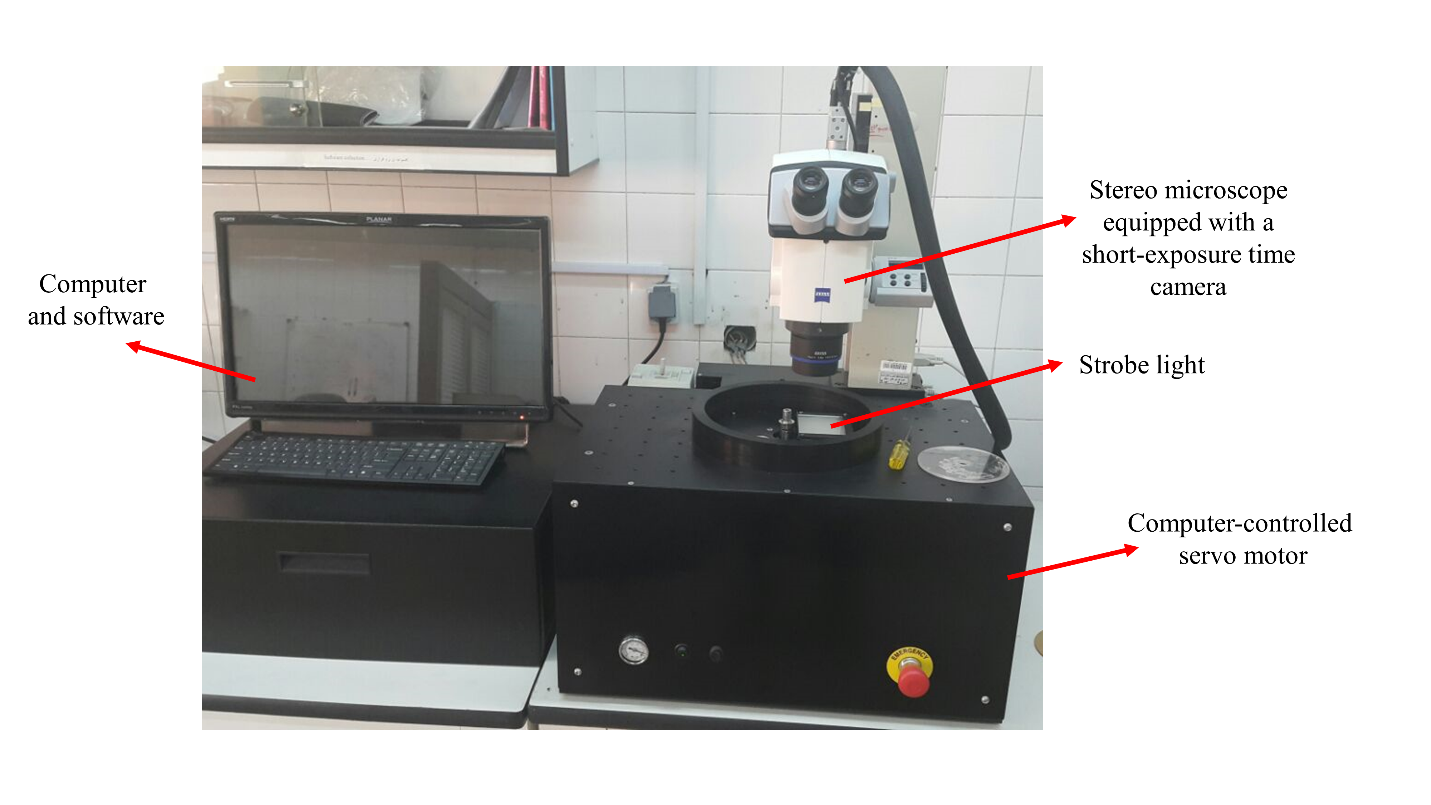


**Fig. S1.** A photograph of the CD-imager setup used for hydrodynamic tests


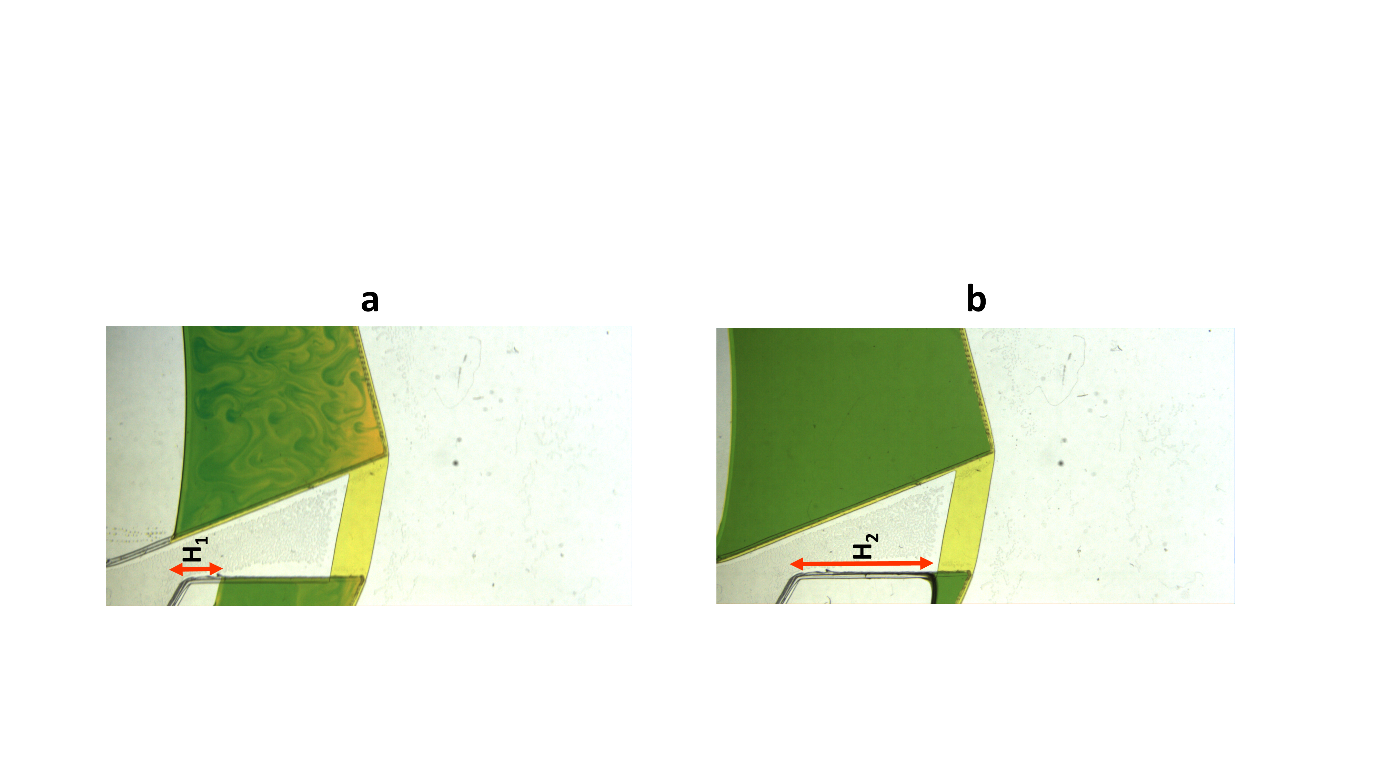


**Fig. S2**. The effect of pneumatic force on liquid transfer; (a) 3500 rpm (b) 1000 rpm


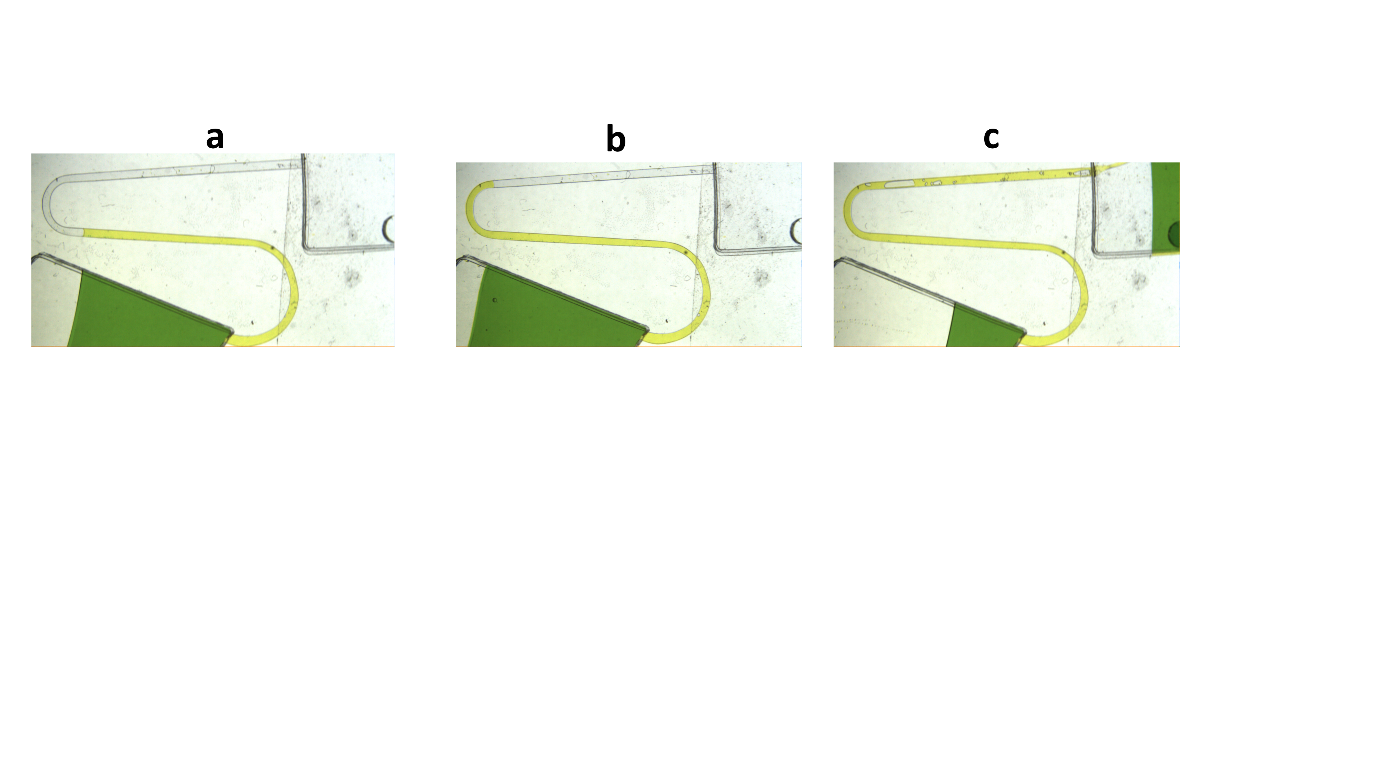


**Fig. S3.** Images of the pneumatic siphon valve performance; (a) the level of the liquid in the siphon microchannel at high rotational frequency, (b) siphon priming at abrupt deceleration after the liquid passing the crest point, (c) withdrawal of the liquid to the collection chamber.


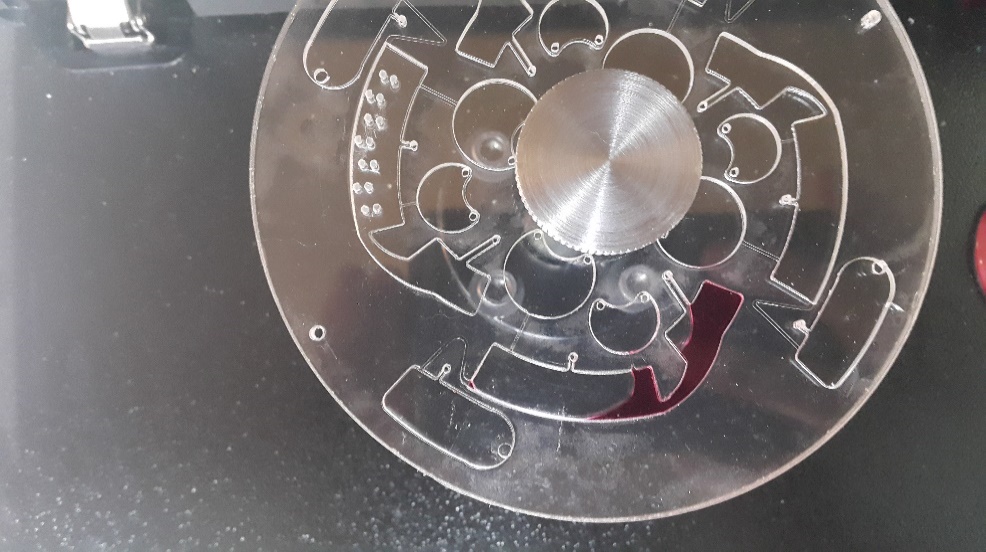


**Fig. S4.** The effect of moment of inertia in mixing process in disc 2.


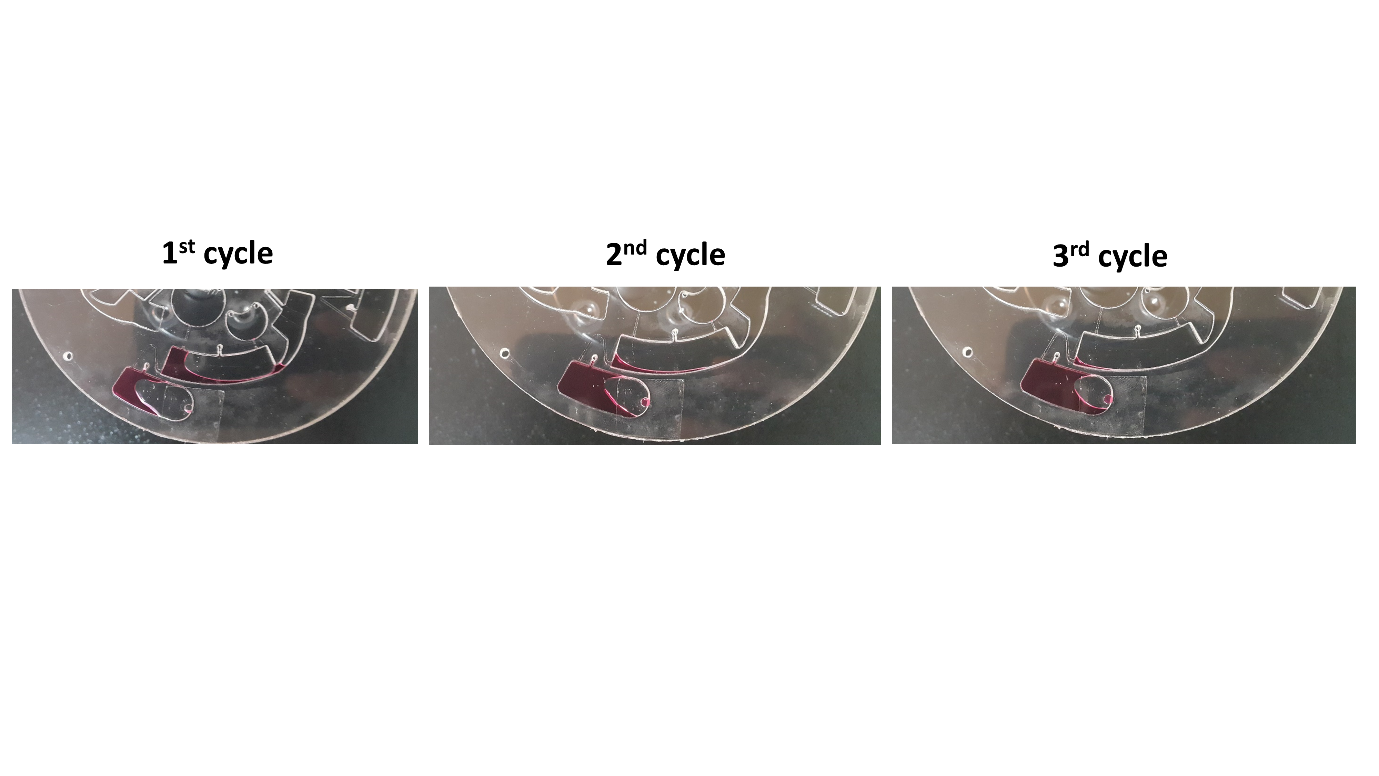


**Fig. S5.** The images of the inertial valve performance.
